# *Notes from the Field:* E-Cigarette and Nicotine Pouch Use Among Middle and High School Students — United States, 2024

**DOI:** 10.15585/mmwr.mm7335a3

**Published:** 2024-09-05

**Authors:** Eunice Park-Lee, Ahmed Jamal, Hannah Cowan, Michael D. Sawdey, Maria R. Cooper, Jan Birdsey, Andrenita West, Karen A. Cullen

**Affiliations:** ^1^Center for Tobacco Products, Food and Drug Administration, Silver Spring, Maryland; ^2^Office on Smoking and Health, National Center for Chronic Disease Prevention and Health Promotion, CDC.

**Summary**

**What is already known about this topic?**

E-cigarettes remain the most used tobacco product among U.S. youths. The wide availability and growing sales of nicotine pouches has also raised concerns about potential use of these products among youths.

**What is added by this report?**

During 2023–2024, current e-cigarette use among middle and high school students declined from 7.7% to 5.9%. Current nicotine pouch use (1.8%) did not change significantly during this period.

**What are the implications for public health?**

Youth e-cigarette use has declined; however, comprehensive tobacco control strategies, regulations, and enforcement remain critical to preventing and reducing e-cigarette and nicotine pouch use among youths.

Current e-cigarette use among U.S. youth has declined considerably since 2019*; however, approximately 2.13 million youths used e-cigarettes in 2023 (*1*). As sales of nicotine pouches (small, dissolvable, flavored pouches containing nicotine derived from tobacco that users place in the mouth between the lip and gum)^†^ have continued to rise nationally since 2016, their use among U.S. youths has become concerning (*2*,*3*). All pouches and most e-cigarettes contain nicotine,^§^ which is highly addictive and can harm the developing adolescent brain (*4*,*5*).

## Investigation and Outcomes

The Food and Drug Administration and CDC analyzed nationally representative data from the 2024 National Youth Tobacco Survey (NYTS), a cross-sectional, school-based, self-administered web-based survey of U.S. students in middle school (grades 6–8) and high school (grades 9–12), which was conducted among 29,861 students from 283 schools during January 22–May 22, 2024.^¶^ Current (i.e., past–30-day) use of e-cigarettes and nicotine pouches was assessed overall, and by frequency of use, device type used for e-cigarettes, any brand and usual brand used,** and flavor types. Weighted prevalence estimates, 95% CIs, and population totals were calculated using SAS-callable SUDAAN software (version 11.0.4; RTI International).^††^ Changes in current use since 2023 were evaluated using *t-*tests; p-values <0.05 were considered statistically significant. The 2023 NYTS data collection methods and estimates have been published (*1*). This activity was reviewed by CDC, deemed not research, and was conducted consistent with applicable federal law and CDC policy.^§§^

In 2024, 5.9% of middle and high school students reported current e-cigarette use, including 7.8% of high school students and 3.5% of middle school students (Table). Among students who currently used e-cigarettes, 38.4% reported frequent use,^¶¶^ and 26.3% reported daily use. The device types used most often by students reporting current e-cigarette use were disposables (55.6%), followed by prefilled or refillable pods or cartridges (15.6%) and tanks or mod systems*** (7.0%); 21.8% of students currently using e-cigarettes were unsure of the device type used. Among students who currently used e-cigarettes, 36.1% used Elf Bar, followed by Breeze (19.9%), Mr. Fog (15.8%), Vuse (13.7%), and JUUL (12.6%); 87.6% used a flavored product; fruit (62.8%), candy (33.3%), and mint (25.1%) were the flavor types most frequently reported.

**TABLE:** Number and percentage of middle and high school students reporting current (past–30-day) e-cigarette use and nicotine pouch use,* overall and by selected characteristics and school level — National Youth Tobacco Survey, United States, 2024

Characteristic	Overall		High school		Middle school	
	Estimated no. of users	Weighted % (95% CI)	Estimated no. of users	Weighted % (95% CI)	Estimated no. of users	Weighted % (95% CI)
**E-cigarette use^†^**						
Current e-cigarette use among all students	1,630,000	5.9 (5.3–6.6)	1,210,000	7.8 (6.9–8.8)	410,000	3.5 (2.9–4.2)
**Among students currently using e-cigarettes**						
**Frequency of use during the previous 30 days^§^**						
1–5 days	720,000	44.1 (40.1–48.1)	510,000	42.3 (37.7–47.1)	200,000	49.7 (43.9–55.6)
6–19 days	280,000	17.5 (15.5–19.6)	180,000	15.5 (13.3–18.0)	90,000	23.5 (20.0–27.3)
20–30 days	620,000	38.4 (34.5–42.5)	510,000	42.1 (37.7–46.7)	110,000	26.8 (21.5–32.8)
**Daily e-cigarette use^§^**	430,000	26.3 (23.0–30.0)	360,000	29.7 (25.9–33.8)	60,000	15.6 (11.5–20.7)
**Device type most often used^¶^**						
Disposables	870,000	55.6 (52.4–58.8)	690,000	58.7 (54.9–62.4)	180,000	47.0 (41.7–52.4)
Prefilled or refillable pods or cartridges	240,000	15.6 (13.5–18.0)	170,000	15.1 (12.9–17.6)	60,000	17.1 (12.7–22.8)
Tanks or mod systems	110,000	7.0 (5.7–8.6)	80,000	7.0 (5.5–8.9)	20,000	6.6 (4.4–9.8)
Don’t know the type	340,000	21.8 (19.4–24.4)	220,000	19.2 (16.5–22.3)	110,000	29.3 (25.0–34.0)
Any brand**						
Elf Bar	560,000	36.1 (32.8–39.6)	380,000	33.2 (29.3–37.3)	170,000	44.4 (39.3–49.6)
Breeze	310,000	19.9 (15.3–25.5)	220,000	19.0 (13.6–26.0)	80,000	21.7 (16.5–28.1)
Mr. Fog	240,000	15.8 (9.6–24.7)	190,000	16.5 (8.9–28.5)	40,000	12.5 (8.9–17.3)
Vuse	210,000	13.7 (10.8–17.2)	160,000	14.2 (10.8–18.4)	40,000	11.3 (7.5–16.5)
JUUL	190,000	12.6 (10.6–14.9)	110,000	10.1 (8.3–12.3)	70,000	19.0 (14.5–24.4)
Esco Bars	160,000	10.2 (8.3–12.4)	100,000	8.9 (6.9–11.4)	50,000	13.0 (9.5–17.4)
Fume	140,000	9.1 (7.2–11.4)	90,000	7.7 (5.8–10.2)	40,000	11.9 (8.1–17.1)
SMOK (including NOVO)	120,000	7.7 (6.2–9.6)	80,000	7.2 (5.6–9.3)	30,000	7.9 (5.4–11.6)
Kangvape (including Onee Stick)	120,000	7.6 (5.9–9.9)	70,000	6.6 (4.7–9.3)	30,000	9.3 (6.7–12.8)
blu	100,000	6.9 (5.6–8.4)	50,000	5.0 (3.8–6.5)	40,000	11.5 (8.6–15.1)
NJOY	90,000	6.1 (4.8–7.7)	60,000	5.5 (4.2–7.3)	20,000	6.7 (4.4–10.0)
Geek Bar^††^	90,000	5.8 (4.3–7.8)	70,000	6.5 (4.7–8.9)	—^§§^	—
Suorin (including Air Bar)	80,000	5.2 (4.1–6.6)	40,000	4.3 (3.2–5.7)	20,000	6.9 (4.9–9.8)
HQD	70,000	5.0 (3.9–6.3)	40,000	3.8 (2.9–5.2)	20,000	7.2 (4.7–10.7)
Logic	70,000	4.9 (3.8–6.3)	40,000	3.9 (2.9–5.2)	20,000	6.9 (4.5–10.4)
Lost Mary^††^	50,000	3.4 (2.4–4.9)	40,000	3.5 (2.3–5.3)	—	—
Some other brand not listed	320,000	20.6 (17.9–23.4)	240,000	20.9 (17.7–24.5)	70,000	18.6 (15.4–22.2)
Not sure or don’t know the brand	490,000	31.1 (28.2–34.2)	350,000	30.6 (27.0–34.4)	120,000	32.6 (28.7–36.8)
**Usual brand^¶¶^**						
Elf Bar	240,000	15.9 (13.1–19.2)	160,000	14.0 (10.7–18.1)	80,000	22.0 (17.6–27.0)
Breeze	130,000	8.7 (5.2–14.2)	100,000	9.0 (5.0–15.7)	—	—
JUUL	50,000	3.2 (2.4–4.4)	20,000	2.5 (1.6–3.8)	20,000	5.4 (3.7–7.8)
Vuse	40,000	3.1 (1.8–5.3)	—	—	—	—
Fume	20,000	1.8 (1.1–3.0)	20,000	2.1 (1.2–3.7)	—	—
Geek Bar^††^	20,000	1.5 (0.9–2.3)	10,000	1.7 (1.0–2.7)	—	—
Esco Bars	20,000	1.4 (0.9–2.2)	10,000	1.1 (0.6–1.9)	—	—
SMOK (including NOVO)	20,000	1.3 (0.8–2.3)	10,000	1.6 (0.9–2.9)	—	—
blu	10,000	1.1 (0.7–1.9)	—	—	—	—
Lost Mary^††^	10,000	0.9 (0.5–1.5)	—	—	—	—
HQD	—	—	—	—	—	—
Kangvape (including Onee Stick)	—	—	—	—	—	—
Logic	—	—	—	—	—	—
Mr. Fog	—	—	—	—	—	—
NJOY	—	—	—	—	—	—
Suorin (including Air Bar)	—	—	—	—	—	—
No usual brand	90,000	6.1 (4.9–7.5)	70,000	6.1 (4.7–7.9)	20,000	6.1 (4.3–8.5)
Some other brand not listed	310,000	20.2 (17.6–23.1)	250,000	21.6 (18.4–25.1)	60,000	16.0 (12.7–19.9)
Not sure or don’t know the brand	420,000	27.1 (24.0–30.5)	310,000	27.3 (23.5–31.5)	100,000	26.7 (22.6–31.2)
Flavored e-cigarette use***						
Any flavor other than tobacco-flavored or unflavored	1,430,000	87.6 (85.2–89.7)	1,070,000	88.2 (85.2–90.7)	350,000	85.7 (81.1–89.3)
Exclusive use of tobacco-flavored or unflavored	100,000	6.4 (5.1–7.8)	70,000	6.1 (4.7–7.8)	30,000	7.3 (4.9–10.8)
Unspecified	90,000	6.0 (4.6–7.9)	60,000	5.7 (4.0–8.1)	20,000	7.0 (4.9–9.9)
**Flavor type used among students currently using e-cigarettes^†††^**						
Fruit	960,000	62.8 (60.0–65.4)	710,000	62.3 (59.0–65.5)	240,000	64.2 (58.6–69.4)
Candy, desserts, or other sweets	510,000	33.3 (30.5–36.3)	360,000	32.2 (28.6–36.0)	140,000	36.4 (32.0–41.0)
Mint	380,000	25.1 (22.3–28.1)	310,000	27.7 (24.2–31.4)	60,000	17.3 (13.5–22.0)
Menthol	230,000	15.1 (12.1–18.7)	190,000	17.0 (13.2–21.6)	30,000	9.5 (6.4–13.9)
Nonalcoholic drinks^§§§^	170,000	11.6 (9.9–13.6)	130,000	11.8 (9.9–14.1)	40,000	10.6 (7.8–14.3)
Unflavored	170,000	11.4 (9.6–13.5)	120,000	11.0 (8.8–13.6)	40,000	12.4 (9.2–16.6)
Alcoholic drinks^§§§^	130,000	8.9 (7.2–10.9)	90,000	8.6 (6.5–11.2)	30,000	9.2 (6.5–12.9)
Tobacco-flavored	130,000	8.5 (7.0–10.3)	70,000	6.8 (5.4–8.7)	50,000	13.1 (9.5–17.8)
Spice^§§§^	90,000	6.4 (5.3–7.8)	60,000	5.9 (4.7–7.4)	20,000	7.2 (4.8–10.7)
Chocolate	80,000	5.8 (4.5–7.4)	50,000	4.7 (3.3–6.4)	30,000	8.1 (5.8–11.3)
Some other flavor	100,000	7.1 (5.7–8.7)	70,000	6.9 (5.3–9.0)	20,000	7.0 (4.7–10.3)
**Use of any flavors that included the word “ice” or “iced” (such as “blueberry ice” or “strawberry ice”)^¶¶¶^**						
Yes	850,000	54.6 (51.5–57.7)	620,000	53.8 (49.8–57.7)	220,000	56.8 (52.1–61.3)
No	490,000	31.8 (29.2–34.6)	380,000	33.5 (30.2–37.0)	100,000	27.4 (23.8–31.4)
Don’t know	210,000	13.6 (11.8–15.5)	140,000	12.7 (10.8–14.9)	60,000	15.8 (12.4–19.9)
Use of any concept flavors with a name that did not describe a specific flavor (such as “solar,” “purple,” “jazz,” “island bash,” or “fusion”)****						
Yes	310,000	20.4 (18.4–22.7)	230,000	20.2 (17.8–22.8)	70,000	20.6 (17.3–24.3)
No	750,000	49.0 (46.0–52.0)	580,000	50.8 (47.1–54.4)	170,000	44.3 (40.2–48.4)
Don’t know	470,000	30.6 (27.7–33.6)	330,000	29.0 (25.5–32.8)	130,000	35.1 (31.4–39.1)
**Nicotine pouch use^††††^**						
Current nicotine pouch use among all students	480,000	1.8 (1.5–2.1)	360,000	2.4 (2.0–2.9)	110,000	1.0 (0.8–1.2)
**Among students currently using nicotine pouches**						
**Frequency of use during the previous 30 days^§^**						
1–5 days	250,000	53.7 (47.9–59.3)	190,000	55.2 (48.6–61.6)	50,000	49.9 (37.9–61.9)
6–19 days	80,000	17.1 (13.4–21.5)	50,000	15.0 (10.8–20.5)	20,000	22.6 (15.5–31.7)
20–30 days	140,000	29.3 (24.7–34.3)	100,000	29.8 (24.3–36.0)	30,000	27.5 (18.5–38.8)
**Daily nicotine pouch use^§^**	100,000	22.4 (18.4–27.0)	80,000	22.9 (17.9–28.7)	20,000	20.5 (13.6–29.6)
Any brand use**						
ZYN	320,000	68.7 (62.7–74.1)	270,000	77.6 (71.4–82.7)	40,000	39.8 (30.5–50.0)
on!	60,000	14.2 (11.1–17.9)	40,000	13.8 (10.2–18.4)	10,000	14.8 (9.2–23.0)
Rogue	60,000	13.6 (10.5–17.4)	40,000	14.0 (10.4–18.7)	10,000	11.7 (7.0–19.0)
Velo	50,000	10.7 (8.3–13.8)	30,000	8.5 (6.1–11.8)	10,000	17.1 (11.4–24.9)
Juice Head ZTN	40,000	9.8 (7.6–12.5)	30,000	8.9 (6.5–12.0)	10,000	11.9 (7.4–18.7)
Fre	40,000	9.7 (7.1–13.0)	20,000	8.1 (5.4–11.9)	10,000	13.4 (7.6–22.4)
2one	30,000	7.4 (5.3–10.2)	10,000	5.2 (3.2–8.4)	10,000	12.8 (7.6–20.7)
Some other brand not listed	20,000	4.6 (2.9–7.2)	—	—	<10,000	7.3 (4.3–11.9)
Not sure or don’t know the brand	70,000	15.3 (11.7–19.8)	30,000	10.4 (7.0–15.2)	30,000	29.0 (21.8–37.6)
**Usual brand^¶¶^**						
ZYN	290,000	62.4 (56.8–67.7)	250,000	72.0 (66.5–77.0)	30,000	33.5 (24.3–44.2)
on!	20,000	4.3 (2.5–7.2)	—	—	—	—
Rogue	10,000	3.5 (1.9–6.2)	—	—	—	—
Fre	10,000	3.4 (2.1–5.4)	—	—	—	—
Juice Head ZTN	10,000	3.1 (1.9–5.2)	—	—	—	—
Velo	10,000	3.0 (1.9–4.7)	—	—	—	—
2one	<10,000	1.9 (1.1–3.3)	—	—	—	—
No usual brand	10,000	2.3 (1.3–4.1)	—	—	—	—
Some other brand not listed	—	—	—	—	—	—
Not sure or don’t know the brand	60,000	13.5 (10.3–17.6)	30,000	9.5 (6.2–14.2)	20,000	26.0 (19.7–33.5)
Flavored nicotine pouch use***						
Any flavor other than tobacco-flavored or unflavored	410,000	85.6 (81.5–88.9)	310,000	86.1 (81.1–89.9)	90,000	85.4 (78.6–90.3)
Exclusive use of tobacco-flavored or unflavored	40,000	9.9 (7.2–13.5)	30,000	10.0 (6.8–14.6)	10,000	9.4 (5.5–15.7)
Unspecified	20,000	4.5 (2.9–6.9)	10,000	3.9 (2.1–6.9)	—	—
**Flavor type used among students currently using nicotine pouches^†††^**						
Mint	240,000	53.3 (47.4–59.1)	200,000	58.8 (52.5–64.8)	30,000	36.8 (25.5–49.6)
Fruit	100,000	22.4 (17.9–27.6)	70,000	20.2 (15.1–26.6)	20,000	27.7 (19.9–37.1)
Menthol	80,000	19.3 (15.1–24.3)	70,000	21.1 (16.1–27.2)	10,000	14.7 (9.0–23.1)
Unflavored	60,000	13.3 (10.0–17.5)	40,000	13.8 (9.9–18.9)	10,000	11.8 (7.3–18.5)
Spice^§§§^	40,000	10.2 (7.5–13.7)	20,000	8.5 (5.6–12.5)	10,000	16.2 (10.1–25.1)
Candy, desserts, or other sweets	40,000	9.5 (7.3–12.2)	20,000	7.8 (5.6–10.6)	10,000	15.7 (10.0–23.8)
Chocolate	30,000	8.1 (5.9–10.9)	20,000	5.8 (4.0–8.4)	10,000	14.4 (8.6–23.3)
Tobacco-flavored	30,000	8.0 (5.9–10.7)	20,000	7.2 (5.0–10.2)	10,000	11.3 (6.7–18.2)
Nonalcoholic drinks^§§§^	30,000	7.5 (5.3–10.5)	20,000	6.6 (4.2–10.4)	10,000	10.5 (6.0–17.7)
Alcoholic drinks^§§§^	30,000	6.6 (4.5–9.6)	10,000	5.5 (3.4–8.8)	<10,000	9.1 (5.2–15.4)
Some other flavor	40,000	9.6 (7.1–13.0)	20,000	6.6 (4.2–10.1)	10,000	17.1 (11.8–24.1)
**Use of any flavors that included the word “ice” or “iced” (such as “blueberry ice” or “strawberry ice”)^¶¶¶^**						
Yes	100,000	23.3 (19.8–27.2)	60,000	19.8 (16.0–24.2)	30,000	34.2 (26.0–43.6)
No	250,000	55.9 (50.8–60.9)	210,000	62.0 (56.0–67.7)	40,000	37.7 (29.3–46.9)
Don’t know	90,000	20.8 (16.9–25.2)	60,000	18.2 (13.8–23.7)	30,000	28.0 (20.5–37.0)
Use of any concept flavors with a name that did not describe a specific flavor (such as “solar,” “purple,” “jazz,” “island bash,” or “fusion”)****						
Yes	50,000	11.4 (8.5–15.3)	20,000	8.6 (5.5–13.3)	20,000	20.9 (14.3–29.5)
No	290,000	64.4 (59.3–69.2)	230,000	70.2 (64.0–75.8)	40,000	46.2 (37.0–55.8)
Don’t know	100,000	24.1 (20.0–28.8)	70,000	21.2 (16.4–26.8)	30,000	32.9 (25.7–40.9)

* Current use of e-cigarettes or nicotine pouches was determined by asking, “During the past 30 days, on how many days did you use [e-cigarettes/a nicotine pouch]?” Current use was defined as use on ≥1 day during the previous 30 days.

^†^ Estimated number of students was rounded down to the nearest 10,000 persons. Subgroup estimates might not sum to overall population estimates because of rounding or exclusion of students who currently used e-cigarettes and who did not report grade level (154), device type (61), any brand (65), usual brand (77), flavor types used (105), use of flavor including the word “ice” or “iced” (83), or use of flavors without specific flavor descriptor (97).

^§^ Frequent use was defined as use on ≥20 days during the previous 30 days. Daily use was defined as use during all of the previous 30 days. These estimates are not mutually exclusive.

^¶^ Device type was ascertained by response to the question, “Which of the following best describes the type of e-cigarette you have used in the past 30 days? If you have used more than one type, please think about the one you use most often.”

** Students currently using e-cigarettes or nicotine pouches were asked, “During the past 30 days, what [e-cigarette/nicotine pouch] brands did you use? (Select one or more).” Those who selected “some other brand(s) not listed here” could provide a write-in response. Write-in responses corresponding to an original response option were recoded.

^††^ Geek Bar and Lost Mary were not included in the list of prespecified response options but were the two most common write-in responses for “some other brand(s) not listed here.” Estimates for Geek Bar and Lost Mary might be underestimated.

^§§^ Data were statistically unreliable because of an unweighted denominator <50 or a relative SE >30%.

^¶¶^ If a student currently using e-cigarettes or nicotine pouches reported a single brand when asked, “During the past 30 days, what [e-cigarette/nicotine pouch] brands did you use (Select one or more),” it was reported as the usual brand. Those who selected two or more brands were asked, “During the past 30 days, what brand of [e-cigarettes/nicotine pouches] did you usually use? (Choose only one answer).” Write-in responses of “some other brand(s) not listed here” were recoded to a corresponding original response option.

*** Students currently using e-cigarettes or nicotine pouches were asked, “In the past 30 days when you used [e-cigarettes/nicotine pouches], what flavors did you use? (Select one or more)?” Those who provided no valid responses were classified as using “unspecified” flavors.

^†††^ Flavor type was ascertained by response to the question, “In the past 30 days when you used [e-cigarettes/nicotine pouches], what flavors did you use? (Select one or more).” Those who selected “some other flavor not listed here” could provide a write-in response; write-in responses corresponding to an original response option were recoded.

^§§§^ These flavor options provided examples: “alcoholic drinks (such as wine, margarita, or other cocktails)”; “non-alcoholic drinks (such as coffee, soda, lemonade, or other beverage)”; and “spice (such as cinnamon, vanilla, or clove).”

^¶¶¶^ Students currently using e-cigarettes or nicotine pouches were asked, “Did any of the flavors you used in the past 30 days have names or descriptions that included the word ‘ice’ or ‘iced’ (for example, blueberry ice or strawberry ice)?”

**** Students currently using e-cigarettes or nicotine pouches were asked, “Did any of the flavors that you used in the past 30 days have a name that did not describe a specific flavor, such as ‘solar,’ ‘purple,’ ‘jazz,’ ‘island bash,’ ‘fusion,’ or some other word or phrase?”

^††††^ Estimated population number of students was rounded down to the nearest 10,000 persons. The total of subgroup estimates might not sum to overall population estimates because of rounding or exclusion of students who currently used nicotine pouches and who did not report grade level (129), any brand (nine), usual brand (12), flavor types used (24), use of flavor including the word “ice” or “iced” (20), or use of flavors without specific flavor descriptor (33).

In 2024, 1.8% of middle and high school students reported current nicotine pouch use, including 2.4% of high school students and 1.0% of middle school students. Among students who currently used nicotine pouches, 29.3% reported frequent use, and 22.4% reported daily use. Among students reporting current nicotine pouch use, 68.7% used ZYN, followed by on! (14.2%), Rogue (13.6%), Velo (10.7%), and Juice Head ZTN (9.8%); 85.6% used a flavored product: mint (53.3%), fruit (22.4%), and menthol (19.3%) were the flavor types most frequently reported.

From 2023 to 2024, current e-cigarette use declined among middle and high school students overall (from 7.7% to 5.9%; p<0.05) and high school students (from 10.0% to 7.8%; p<0.05). No significant changes were observed for current e-cigarette use among middle school students or for current nicotine pouch use among high school students or middle and high school students overall.

## Conclusions and Actions

In 2024, an estimated 1.63 million U.S. middle and high school students currently used e-cigarettes, a significant decline from 2.13 million in 2023. In contrast, from 2023 to 2024, no significant changes occurred in current nicotine pouch use among middle and high school students overall (an estimated 480,000 students in 2024), despite rising sales of nicotine pouches (*2*).^†††^ Continued surveillance of youth tobacco product use patterns and implementation of comprehensive tobacco control strategies, regulations, and enforcement^§§§^ are important for preventing and reducing tobacco product use by youths and associated adverse health outcomes, including a potential lifetime of nicotine addiction.

## Footnotes

*
http://dx.doi.org/10.15585/mmwr.ss6812a1

† Unlike other smokeless tobacco products, such as snuff and snus, nicotine pouches do not contain any tobacco leaf.

§ E-cigarettes: https://www.fda.gov/tobacco-products/products-ingredients-components/e-cigarettes-vapes-and-other-electronic-nicotine-delivery-systems-ends; nicotine pouches: https://www.fda.gov/tobacco-products/products-ingredients-components/other-tobacco-products.

¶ In 2024, the student-level participation rate was 78.3%, and the school-level participation rate was 42.7%, for an overall response rate of 33.4%.

** Brand response options were: blu, Breeze, Elf Bar, Esco Bars, Fume, JUUL, HQD, Kangvape (including Onee Stick), Logic, Mr. Fog, NJOY, SMOK (including NOVO), Suorin (including Air Bar), Vuse, “some other brand(s) not listed here,” and “I don’t know the brand.” Those who selected “some other brand(s) not listed here” could provide a write-in response. Write-in responses were recoded into valid responses. Estimates for Geek Bar and Lost Mary were based on the write-in responses and might be underestimated.

†† Data were weighted to account for complex survey design and to adjust for nonresponse. The weighted proportions of students in each grade matched national population proportions for U.S. public and private schools derived from data from the National Center for Education Statistics (2021–2022 Common Core of Data and 2019–2020 Private School Universe Study) and Market Data Retrieval, Inc. Population number estimates were rounded down to the nearest 10,000 students.

§§ 45 C.F.R. part 46.102(l)(2), 21 C.F.R. part 56; 42 U.S.C. Sect. 241(d); 5 U.S.C. Sect. 552a; 44 U.S.C. Sect. 3501 et seq.

¶¶ Frequent use was defined as use on ≥20 days during the previous 30 days. Daily use was defined as use on all of the previous 30 days. These estimates are not mutually exclusive.

*** An e-cigarette with a tank that the user refills with liquids or a mod system that can be customized by the user with their own combination of batteries or other parts.

††† The scope of the current report examined changes in prevalence from 2023 to 2024. Although outside the scope of this report, a small but statistically significant increase in current nicotine pouch use occurred from 2022 to 2024. The 2022 NYTS methodology report and a copy of the 2022 data file can be found at https://www.cdc.gov/tobacco/about-data/surveys/national-youth-tobacco-survey.html.

§§§ https://www.cdc.gov/tobacco/php/state-and-community-work/guides-for-states.html?CDC_AAref_Val=https://www.cdc.gov/tobacco/stateandcommunity/guides/index.htm; https://www.fda.gov/tobacco-products/ctp-newsroom/fdas-comprehensive-plan-tobacco-and-nicotine-regulation
